# Glycaemia and critical care outcomes

**DOI:** 10.1186/cc13631

**Published:** 2014-03-17

**Authors:** M Cecconi, C Ryan, D Dawson, N Di Tomasso, G McAnulty, B Philips, A Rhodes

**Affiliations:** 1 St George's Hospital, London, UK; 2 St George's Healthcare NHS Trust, London, UK

## Introduction

The aim of this study was to determine the impact of preadmission or first 24-hour blood glucose (BG) measurements in UK ICUs on mortality.

## Methods

The Intensive Care National Audit & Research Centre case-mix programme database on adult admissions to general, neuroscience and cardiothoracic critical care units was used for analysis. Within the database, the highest and lowest blood glucose (BG) values measured during the first 24 hours from admission were recorded. BG control value was defined as BG (≥4.0 ≤9.9 mmol/l). Other BG levels were defined as: very low, ≤2.2 mmol/l (≤40 mg/dl); low, >2.2 ≤3.9 mmol/l (40 to 70 mg/dl); high, ≥10.0 <11.1 mmol/l (180 to 200 mg/dl); and very high, ≥11.1 mmol/l (200 mg/dl).

**Table 1:** Critical care outcomes

Variable	Very low	Low	Control	High	Very high
Number of patients	11,471	70,106	521,839	77,423	187,553
ICU mortality* (%)	55	31	13	18	26
ICU LOS*	7	6	4	5	6

*P <0.001.

## Results

There was an increased incidence of mortality in those patients with at least one BG measure below 3.9 mmol/l (70 mg/dl) compared with those without (Table 1). There was a link between BG levels and LoS for surviving patients with the longest hospital stays (critical care and total hospital) experienced by those with BG levels below 2.2 mmol/l (40 mg/dl).

## Conclusion

There is a strong association between BG levels during admission and mortality and LoS outcomes. Although it is not possible to make the link with causation from our dataset, we present results from the largest single dataset of critical care unit patients [1,2].
